# Increased PUFA Content and 5-Lipoxygenase Pathway Expression Are Associated with Subcutaneous Adipose Tissue Inflammation in Obese Women with Type 2 Diabetes

**DOI:** 10.3390/nu7095362

**Published:** 2015-09-11

**Authors:** Mattijs M. Heemskerk, Martin Giera, Fatiha el Bouazzaoui, Mirjam A. Lips, Hanno Pijl, Ko Willems van Dijk, Vanessa van Harmelen

**Affiliations:** 1 Department of Human Genetics, Leiden University Medical Center, Leiden 2300 RC, The Netherlands; E-Mails: m.m.heemskerk@lumc.nl (M.M.H.); f.el_bouazzaoui@lumc.nl (F.B.); kowvd@lumc.nl (K.W.V.D.); 2 Einthoven Laboratory for Experimental Vascular Medicine, Leiden University Medical Center, Leiden 2300 RC, The Netherlands; 3 Center of Proteomics and Metabolomics, Leiden University Medical Center, Leiden 2300 RC, The Netherlands; E-Mail: m.a.giera@lumc.nl; 4 Department of Medicine, division Endocrinology, Leiden University Medical Center, Leiden 2300 RC, The Netherlands; E-Mails: m.a.lips@lumc.nl (M.A.L.); h.pijl@lumc.nl (H.P.)

**Keywords:** leukotrienes, arachidonic acid, omega-3 fatty acids, lipidomics, gene expression

## Abstract

Obese women with type 2 diabetes mellitus (T2DM) have more inflammation in their subcutaneous white adipose tissue (sWAT) than age-and-BMI similar obese women with normal glucose tolerance (NGT). We aimed to investigate whether WAT fatty acids and/or oxylipins are associated with the enhanced inflammatory state in WAT of the T2DM women. Fatty acid profiles were measured in both subcutaneous and visceral adipose tissue (vWAT) of 19 obese women with NGT and 16 age-and-BMI similar women with T2DM. Oxylipin levels were measured in sWAT of all women. Arachidonic acid (AA) and docosahexaenoic acid (DHA) percentages were higher in sWAT, but not vWAT of the T2DM women, and AA correlated positively to the gene expression of macrophage marker *CD68*. We found tendencies for higher oxylipin concentrations of the 5-LOX leukotrienes in sWAT of T2DM women. Gene expression of the 5-LOX leukotriene biosynthesis pathway was significantly higher in sWAT of T2DM women. In conclusion, AA and DHA content were higher in sWAT of T2DM women and AA correlated to the increased inflammatory state in sWAT. Increased AA content was accompanied by an upregulation of the 5-LOX pathway and seems to have led to an increase in the conversion of AA into proinflammatory leukotrienes in sWAT.

## 1. Introduction

Obesity is closely associated with insulin resistance, type-2 diabetes mellitus (T2DM), dyslipidemia, hypertension, and cardiovascular disease. Expanding adipose tissue plays an important role in the pathophysiology of obesity-associated disorders as it responds to the energy overload with stress signals which, in turn, can elicit local immune responses and inflammation [1]. Although the majority of obese individuals (~80%) will eventually develop metabolic disorders associated with a reduced life expectancy, there seems to be a subset of obese individuals that remains relatively insulin sensitive and metabolically healthy throughout life [2,3]. The reason why these individuals are unaffected is still not completely understood. We have previously shown that subcutaneous white adipose tissue (sWAT) from obese women with T2DM contained a larger number of crown-like structures (CLS) than sWAT of similarly obese women with normal glucose tolerance (NGT) [4]. In a parallel study, we analyzed the transcriptome in adipose tissue samples by RNA deep sequencing in the same cohort of women and observed an up-regulation of genes in inflammatory pathways and a down-regulation of genes in metabolic pathways in WAT of the women with T2DM [5]. Although metabolic health comprises more components than normal glucose tolerance, our data indicate that metabolically healthy and unhealthy obese women can be differentiated by the inflammatory status of their adipose tissue. 

Adipose tissue inflammation involves the accumulation of macrophages in CLS around adipocytes that are stressed or dying due to cellular lipid overload [6]. The enlarged stressed adipocytes exhibit an increased release of pro-inflammatory adipocytokines and chemokines which attract immune cells into the adipose tissue [7]. Hypertrophic adipocytes also store and release increased levels of fatty acids which are known to mediate inflammatory processes as well [8]. Not only the amount of fatty acids released, but also the type of fatty acid stored in adipose tissue, has been associated to systemic inflammation [9] and T2DM [10,11]. For example, an increased percentage of saturated fatty acids or their metabolites has been suggested to induce inflammation by activating Toll-like receptor 4, which in turn leads to an up-regulation of the ceramide biosynthesis pathway [12]. Ceramides activate the NLPR3 inflammasome which is an important contributor to obesity induced inflammation and insulin resistance [13]. Other fatty acids that are involved in inflammation are the polyunsaturated fatty acids (PUFAs). *n*-6 and *n*-3 PUFAs can induce pro- and anti-inflammatory pathways respectively [14] via a variety of different mechanisms including signaling via GPR120. PUFAs can be converted to pro- or anti-inflammatory lipid mediators called oxylipins. Oxylipin synthesis usually occurs via the cyclooxygenase (COX), lipoxygenase (LOX) or cytochrome P450 (CYP) pathways [15]. All three pathways can metabolize both *n*-3 and *n*-6 PUFAs, but the affinity for these substrates differs as does the pro- or anti-inflammatory potency of the different resulting products. For example the 2-series prostaglandins, synthesized from arachidonic acid (AA; 20:4*n*-6) via the COX pathway, induce mainly pro-inflammatory effects; while the 3-series prostaglandins, synthesized from eisosapentaenoic acid (EPA; 20:5*n*-3) via COX, induces less potent inflammatory effects [16] and the epoxy metabolites derived from *n*-3 PUFAs via the CYP pathway induce anti-inflammatory effects [17]. PUFA-derived oxylipin synthesis can be rapidly induced when triggered by inflammasome signaling [18]. A wide range of oxylipins has been identified in both human [19] and mouse WAT [20]. Oxylipins have been shown to play a role in WAT inflammation, mostly in rodent studies [8]. In the current study we hypothesized that the adipose fatty acid composition is linked to the enhanced inflammatory state in adipose tissue of obese women with T2DM. To test this hypothesis, we determined fatty acid profiles by GC-MS and oxylipin profiles by LC-MS/MS in white adipose tissue of both obese women with T2DM and obese women with NGT.

## 2. Experimental Section

### 2.1. Subjects

The study group consisted of 19 obese women with normal fasting glucose and normal glucose tolerance (NGT) and 16 women with T2DM, as tested by fasting glucose levels and a mixed meal tolerance test in a previous study [21]. The women were part of a clinical trial of which the research methods and design have been described by Lips *et al.* [21]. The groups were comparable for age and BMI (see Table 1). All women had been morbidly obese (mean BMI = 43.4 ± 3.8 kg/m^2^) for at least five years. Women who reported the use of weight loss medications within 90 days prior to enrolment in the study were excluded. Body weight of all women had been stable for at least three months prior to inclusion. All women were non-smokers, had no signs of any infections, nor had any history of auto-immune diseases. Before inclusion into the study, subjects were asked to fill out questionnaires consisting of questions on diets and eating habits, and to keep a detailed food intake diary during one week. There were no significant differences in meat, fish or dairy intake between the subject groups. The women underwent bariatric surgery (gastric bypass or banding). Within 1 h after opening the abdominal wall adipose tissue specimens were taken from the epigastric region of the abdominal wall (subcutaneous sWAT) and from the major omentum (visceral vWAT). These samples were used for determination of fatty acid composition and oxylipin profiles. The study (ClinicalTrials.gov: NTC01167959) was approved by the Ethics Committee of Leiden University. All subjects gave informed consent to participate in the study.

### 2.2. Medication

For obvious reasons we could not restrict obese individuals to not using any type of medication. All T2DM women were treated with oral medication only (metformin or sulfonylurea derivatives). Participants were allowed to use cholesterol-lowering statins and antihypertensive medication.

The use of drugs such as statins and antihypertensive drugs was slightly higher in the T2DM women. The patients were not using any anti-inflammatory agents (*i.e.*, NSAIDS, thiazolidinediones or steroids (prednisone)).

### 2.3. Analysis of Number of Crown-Like Structures and Adipocyte Size

In a previous study the number of CLS in the adipose tissue of our participants was determined by immunohistochemistry and the adipocyte size by direct microscopy [4].

### 2.4. Fatty Acid Composition of WAT by GC-MS

FA composition analysis of sWAT and vWAT was carried out as described recently by Kloos *et al.* [22]. Approximately 10 mg WAT was weighed from the obese women. Water (1 mL), methanol (3 mL) and 10 M NaOH (1 mL) were added, the samples flushed with argon and hydrolyzed for 1 h at 90 °C. After acidification with 2 mL of 6 M HCl, 10 µL of an internal standard solution ([^2^H_31_]palmitic acid and ergosterol 10 µg/mL each) was added. The samples were extracted twice with 3 mL *n*-hexane and the combined organic extracts were dried under a gentle stream of nitrogen. Dried samples were derivatized using 25 µL of *N*-*tert*-butyldimethylsilyl-*N*-methyltrifluoroacetamide (Sigma Aldrich, Schnelldorf, Germany) for 10 min at 21 °C, subsequently 25 µL of *N*,*O*-bis(trimethylsilyl)trifluoroacetamide containing 1% trimethylchlorosilane (Thermo Scientific, Waltham, MA, USA) and 2.5 µL of pyridine were added and the sample was heated for 15 min to 50 °C. Next, 947.5 µL of *n*-hexane, containing 10 µg/mL octadecane (C18) as a system monitoring component, was added.

Samples were analyzed in SIM mode on a Scion TQ GC-MS (Bruker, Bremen, Germany) equipped with a 15 m × 0.25 mm × 0.25 mm BR5MS column (Bruker). The injection volume was 1 µL, the injector was operated in splitless mode at 280 °C, and the oven program was as follows: 90 °C kept constant for 0.5 min, then ramped to 180 °C with 30 °C/min then to 250 °C with 10 °C/min then to 266 °C with 2 °C/min, and finally to 300 °C with 120 °C/min, kept constant for 2 min. Helium (99.9990%, Air Products, The Netherlands) was used as the carrier gas. For data analysis a total area correction was applied.

### 2.5. Oxylipin Measurements in WAT by LC-MS/MS

Oxylipin analysis was carried out as described elsewhere ([23,24]) with some modifications. Approximately 100 mg of tissue were cut using a razor blade on a glass plate, transferred into a 2 mL Eppendorf tube and accurately weighed. Three microliters of an internal standard solution containing 50 ng/mL each of PGE_2_-d4, LTB_4_-d4, 15-HETE-d8 and DHA-d5 was added. Subsequently 3–5 stainless steel beads, 500 µL of methanol and 2 µl of a 20 mg/mL butylated hydroxyl toluene solution were added. Next, the samples were homogenized in a bead beater for 4 min centrifuged at 16,100 *g* for 3 min. Four hundred microliters of the supernatant were transferred into a 12 mL glass tube. The samples were re-extracted using 500 µL of methanol by shaking for 5 min. The combined organic extracts were diluted with approximately 9 mL of water, acidified with 6 M HCl and further cleaned up using solid phase extraction (employing 100 mg SPE columns) as described elsewhere [25] and finally reconstituted in 150 µL 40% methanol. LC-MS/MS analysis was done with Multiple Reaction Monitoring detection based on pure standards of the target compounds (see Table S1 for the Multiple Reaction Monitoring settings).

### 2.6. Statistics

Data are expressed as mean ± SD or as median and range of the values. Differences between NGT and T2DM were analyzed using unpaired non-parametric *t*-tests. Linear regression was used to analyze correlations using the *F*-test in Graphpad Prism 6 (GraphPad Software, CA, USA).

## 3. Results

### 3.1. Characteristics of Participants

Characteristics of the participants are shown in Table 1. Fasting plasma glucose was significantly higher and LDL-cholesterol levels were lower in T2DM than in NGT women. HOMA-IR index and triglyceride levels tended to be higher in the T2DM women. The NGT and T2DM had similar waist circumferences, indicating equal upper body obesity. The gene expression of *CD68* (a macrophage marker) and the number of CLS per area adipose tissue on immunohistochemistry slides was used as an index for the extent of adipose tissue inflammation. The sWAT but not the vWAT of the T2DM women had a significantly higher gene expression of *CD68* and contained more CLS. Adipocyte sizes did not differ between NGT and T2DM women both for sWAT and vWAT.

**Table 1:** Characteristics of the normal glucose tolerance (NGT) and type 2 diabetes mellitus (T2DM) women. Crown-like structures (CLS) were determined by immunohistochemistry of CD68 and expressed as number (#) of CLS per area of adipose tissue (AT) section on the slide. Adipocyte size was expressed as mean adipocyte diameter in µm. CLS counts and adipocyte sizes have been published previously [4]. Gene expression was determined by RNA deep sequencing in a previous study [5] and expressed as log transformed normalized gene expression levels (relative units [RU]: log2-scale). Data are expressed as mean ± SD.

	NGT	T2DM	*p*-Value *t*-Test
*N*	19	16	
BMI (kg/m^2^)	43.4 ± 3.3	43.4 ± 4.5	NS
Age (years)	47 ± 7	52 ± 6	NS
Waist circumference (cm)	121 ± 8	127 ± 12	NS
HOMA-IR	2.7 ± 2.2	4.0 ± 3.0	0.08
Fasting glucose (mmol/L)	5.0 ± 0.6	9.0 ± 2.6	<0.01
Fasting insulin (mU/L)	10.9 ± 7.7	13.3 ± 7.3	NS
Total cholesterol (mmol/L)	4.7 ± 1.1	4.2 ± 0.8	NS
HDL cholesterol (mmol/L)	1.1 ± 0.3	1.1 ± 0.3	NS
LDL cholesterol (mmol/L)	3.0 ± 1.0	2.2 ± 0.6	0.03
Triglycerides (mmol/L)	1.5 ± 0.7	2.0 ± 0.7	0.08
CRP (mg/L)	7.8 ± 7.5	8.2 ± 6.3	NS
*CD68* gene expression in sWAT	8.2 ± 0.4	8.9 ± 0.7	0.003
*CD68* gene expression in vWAT	8.2 ± 0.4	8.4 ± 0.5	NS
# of CLS in sWAT (no/AT section)	2.3 ± 1.4	12.3 ± 7.1	0.05
# of CLS in vWAT (no/AT section)	1.9 ± 1.2	2.7 ± 1.0	NS
Adipocyte size in sWAT (µm)	122 ± 16	126 ± 15	NS
Adipocyte size in vWAT (µm)	112 ± 16	118 ± 8	NS

### 3.2. Differences in Adipose Tissue Content of Saturated Fatty Acids and PUFAs between NGT and T2DM Obese Women

**Figure 1:**
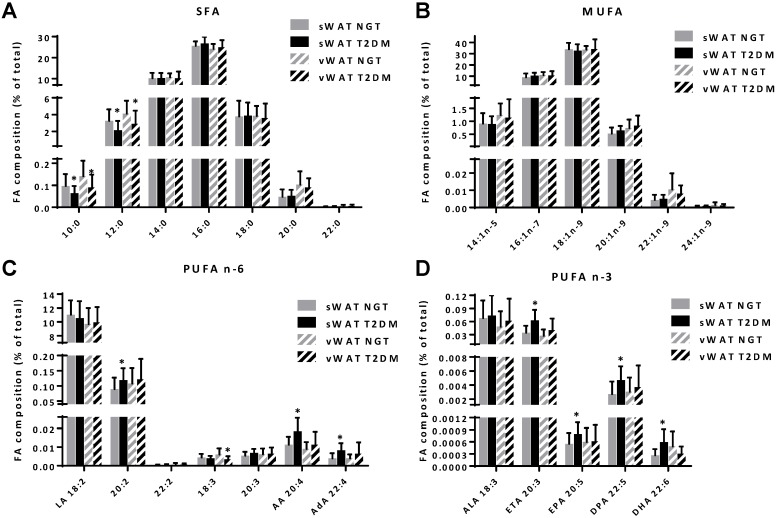
Fatty acid composition of white adipose tissue (sWAT) and visceral adipose tissue (vWAT) for normal glucose tolerance (NGT) versus type 2 diabetes mellitus (T2DM) individuals. (**A**) Saturated fatty acids; (**B**) mono-unsaturated fatty acids; (**C**) *n*-6 and (**D**) *n*-3 poly-unsaturated fatty acids. Data are expressed as mean ± standard deviation (SD). * *p* < 0.05 for NGT *versus* T2DM.

The fatty acid composition of sWAT and vWAT samples from the NGT and T2DM obese women was determined by GC-MS. For both sWAT and vWAT for the T2DM women, we found lower percentages of 10:0 (decanoic acid) and 12:0 (lauric acid) (Figure 1A). The MUFAs did not show large differences between the NGT and T2DM women (Figure 1B). For the PUFAs in vWAT of the T2DM women, a lower percentage of 18:3*n*-6 (γ-linolenic acid) was seen. In the sWAT of the T2DM women, differences in both *n*-6 and *n*-3 PUFAs were found. For the *n*-6 PUFAs higher percentages of 20:4*n*-6 (arachidonic acid) and 22:4*n*-6 (adrenic acid) were found (Figure 1C), whereas for the *n*-3 PUFAs higher percentages of 20:3*n*-3 (eicosatetraenoic acid), 20:5*n*-3 (eicosapentaenoic acid), 22:5*n*-3 (docosapentaenoic acid), and 22:6*n*-3 (docosahexaenoic acid) were found (Figure 1D).

### 3.3. Arachidonic Acid Correlated to CD68 Expression in sWAT

**Figure 2:**
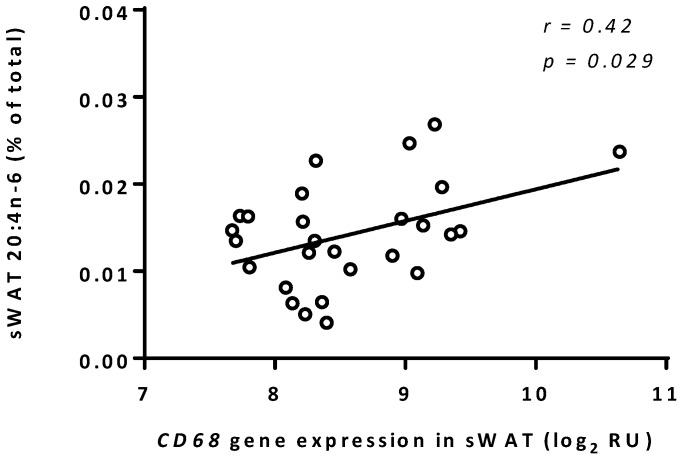
*CD68* expression *versus* AA percentage in white adipose tissue (sWAT). Linear regression of sWAT gene expression of the macrophage marker *CD68* with the sWAT percentage of 20:4*n*-6 (AA). Data are log transformed normalized gene expression levels (relative units [RU]: log2-scale). Data are expressed as mean ±standard deviation (SD).

The percentage AA of the total fatty acid pool correlated to the gene expression level of *CD68* in sWAT. For the correlation of AA with *CD68* expression the goodness-of-fit was *r* = 0.42, *p* = 0.029 (Figure 2). There were no significant correlations between any other fatty acid percentage and *CD68* expression in sWAT. 

### 3.4. Oxylipin Levels in sWAT of Women with NGT and T2DM

**Figure 3:**
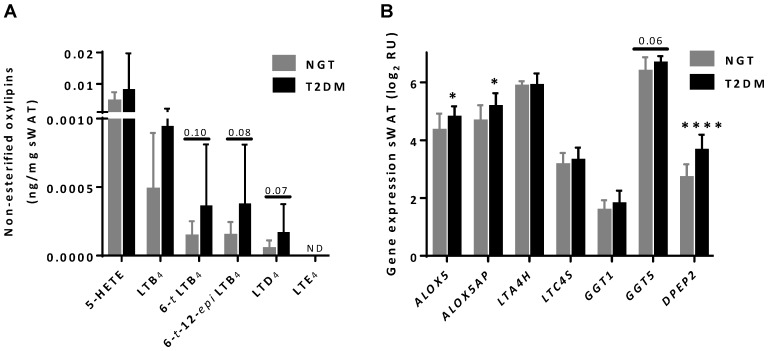
5-LOX oxylipins in white adipose tissue (sWAT). (**A**) Non-esterified oxylipins derived from arachidonic acid via the 5-LOX pathway in sWAT of normal glucose tolerance (NGT) *versus* type 2 diabetes mellitus (T2DM) obese individuals. LTE_4_ was below the detection limit (ND not detectable). LTA_4_, LTC_4_, and LTF_4_ were not included in the LC-MS/MS method. (**B**) Gene expression of genes involved in the leukotriene biosynthesis pathway in sWAT of NGT *versus* T2DM individuals (*ALOX5*, *ALOX5AP*, *LTA4H*, *LTC4S*, *GGT1*, *GGT5*, and *DPEP2*). Data are log transformed normalized gene expression levels (relative units [RU]: log2-scale). Data are expressed as mean ± standard deviation (SD). * *p* < 0.05 and **** *p* < 0.0001 for NGT *versus* T2DM.

Table 2 shows the list of the oxylipins that were measured and the levels that were detected per mg of sWAT of the women with NGT and with T2DM. Many of the oxylipins measured, in particular the resolvins, were below the detection limit (Table 2). There was a large variation in oxylipin concentrations between the women. Of the detectable oxylipins there was a tendency for higher content of some leukotrienes; *i.e.*, LTD_4_, 6-*trans* LTB_4_ and 6-*trans-*12-*epi* LTB_4_ in sWAT of women with T2DM (Figure 3A). Supplementary Figures S1–S3 contain examples of representative chromatograms for 5-HETE, LTD_4_, LTB_4_, 6-*trans* LTB_4_ and 6-*trans-*12-*epi* LTB_4_ and the insert of these figures show the corresponding mass spectrum of the first three compounds. 

### 3.5. Differential Expression of ALOX5, ALOX5AP and DPEP2 in sWAT between NGT and T2DM Women

**Figure 4:**
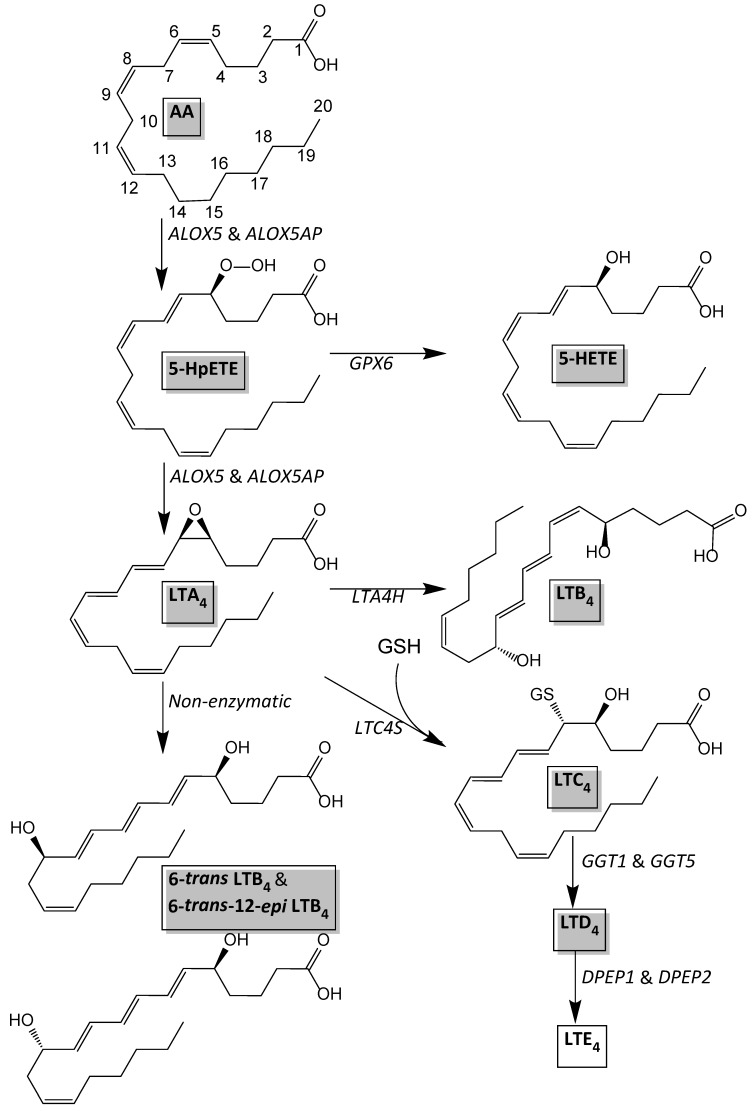
Leukotriene biosynthesis pathway based on Murphy *et al.* [30].

In a previous study, the adipose tissues of 15 of the NGT and 15 of the T2DM women included in the current study were used for transcriptome analysis by RNA deep sequencing, as described in [5]. From the gene expression profiles obtained in that study we could extract gene expression levels of the genes involved in the leukotriene biosynthesis pathway (*i.e.*, *ALOX5*, *ALOX5AP*, *LTA4H*, *LTC4S*, *GGT1,* *GGT5*, and *DPEP2*). Figure 4 describes a scheme for the leukotriene biosynthesis pathway and the genes involved therein. Of these genes *ALOX5*, *ALOX5AP*, and *DPEP2* were expressed significantly higher and *GGT5* tended (*p* = 0.06) to be expressed higher in sWAT of T2DM women (Figure 3B).

**Table 2:** Oxylipins in white adipose tissue (sWAT) of normal glucose tolerance (NGT) *versus* type 2 diabetes mellitus (T2DM) obese women. NGT *versus* T2DM values were compared using non-parametric *t*-test. ND, non-detectable. NS, non-significant. * Compound not in calibration line. *p*-Value calculated from area ratios compared to internal standard. # Detected, but only in fewer than five individuals in total. Data are expressed as median and range of the concentrations.

Oxylipin (pg/mg sWAT)	NGT		T2DM		NGT *vs.* T2DM
	Range	Median	Range	Median	*p*-Value
5-HETE	0.8–32	3.7	1.5–57	5.0	NS
8-HETE	0.5–18	2.3	0.6–22	2.9	NS
11-HETE	0.4–15	1.6	0.4–18	1.9	NS
12-HETE	1.6–84	11	2.8–79	12	NS
15-HETE	0.7–32	3.5	0.8–46	4.0	NS
15-HEPE	0.09–3.1	0.4	0.2–2.6	0.6	NS
18-HEPE	0.1–4.9	0.6	0.2–3.9	0.5	NS
7-HDHA	*		*		NS
10-HDHA	*		*		NS
17-HDHA	0–3.2	0.5	0–3.2	1.1	NS
LTB_4_	0.06–2.7	0.4	0.05–4.2	0.4	NS
6-*trans*-12-*epi* LTB_4_	0.03–0.3	0.1	0.06–1.7	0.2	0.08
6-*trans* LTB_4_	0.04–0.4	0.1	0.06–1.9	0.2	0.10
20-OH LTB_4_	ND		ND		
LTD_4_	0–0.2	0.04	0–0.8	0.08	0.06
LTE_4_	ND		ND		
PGD_2_	0.06–1.1	0.3	0.04–2.7	0.3	NS
PGE_2_	0.07–2.1	0.6	0.08–3.6	0.5	NS
PGF_2α_	0–2.1	0.7	0.1–2.5	0.5	NS
TxB_2_	0–12	1.5	0.05–20	1.6	NS
LXA_4_	0–0.2	0	0–1.5	0.02	NS
LXB_4_	ND		ND		
AT LXA_4_	ND		ND		
8-*iso* PGE_2_	ND		ND		
8-*iso* PGF_2α_	0–0.2	0.05	0–0.8	0.04	NS
15-*keto* PGE_2_	0.02–0.4	0.09	0.006–1.2	0.06	NS
13,14-dihydro-15-*keto* PGF_2α_	0–5.0	0	0–4.6	0	NS
RvD1	ND		ND		
RvD2	ND		ND		
AT RvD1	ND		ND		
RvE1	ND		ND		
RvE2	#		#		
18S-RvE3	ND		ND		
18R-RvE3	ND		ND		
7,17-DiHDPA	0–1.7	0.08	0–0.8	0	NS
19,20-DiHDPA	0–0.3	0.09	0.06–0.4	0.1	NS
10*S*,17*S*-diHDHA (PDX)	0–0.2	0.04	0–0.2	0.04	NS
MaR1	#		#		
7S-MaR1	ND		ND		
5,15-diHETE	0–20	2.2	0–87	3.4	NS
14,15-diHETE	0–0.6	0.2	0–2.2	0.2	NS
8*S*,15*S*-diHETE	0.04–1.3	0.1	0–1.6	0.2	NS
9-HoDE	75–924	317	127–832	259	NS
13-HoDE	47–527	161	65–471	142	NS
9-HoTrE	3.4–49	21	3.9–37	15	NS
13-HoTrE	45–502	285	57–396	195	NS
8(9)EET	*		*		NS
11(12)EET	*		*		NS
14(15)EET	*		*		NS

## 4. Discussion

We have previously shown that the macrophage content in the sWAT, but not the vWAT, of obese women with T2DM was higher. In the current study we investigated whether this increased inflammatory state was linked to a higher percentage of fatty acids that mediate inflammatory processes such as the saturated fatty acids or the *n*-6 PUFAs. We found that the higher inflammatory state in the sWAT of T2DM women was not associated with a higher percentage of saturated fatty acids. Interestingly, the sWAT of T2DM women showed increased percentages of both *n*-3 and *n*-6 long-chain PUFAs (including AA and DHA) which was not seen in vWAT. AA percentage in sWAT correlated positively with gene expression of the macrophage marker *CD68* indicating that AA was associated with the macrophage infiltration in the adipose tissue. 

The content of PUFAs in adipose tissue is dependent on several processes including uptake of PUFAs into the cell via fatty acid transporters or passive transport, the biosynthesis of PUFAs within a cell, the degradation of PUFAs and the release of PUFAs from a cell. Previously, we analyzed the transcriptome of adipose tissue by RNA sequencing in the same individuals as included in this study [5]. By using a network-based approach to analyze gene expression in NGT *versus* T2DM women, we identified the down-regulation of the complete acetyl-CoA metabolic network in the adipose tissue of the T2DM women. This network included a down-regulation of fatty acid biosynthesis, fatty acid degradation as well as fatty acid release. Thus, although there seem to be clear differences in gene expression of PUFA metabolism, we cannot determine the net flux of PUFAs in the adipose tissue based on this gene expression analysis. Further research is required to determine the PUFA mass balance *in vivo* in the adipose tissue to be able to explain the increased PUFA content in the T2DM individuals.

By determining the PUFA content in adipose tissue by lipid hydrolysis, we obtained a total view of all PUFAs present. Although most of the fatty acids in adipose tissue are stored in triacylglycerols within the adipocytes (99%) there is a small fraction of the fatty acids present in phospholipids. We could not determine which of the lipid species in the adipose tissue contributed to the difference in PUFAs in T2DM women. In addition, the (immune) cells of the stromal vascular fraction in adipose tissue contribute to the fatty acid pool and can produce fatty acid-derived mediators. Immune cells are the main producers of free oxylipins [26]. Additional research is necessary to determine the proportion of oxylipin content in adipose tissue produced by the adipocytes and the cells in the stromal vascular fraction.

Both AA and DHA can be metabolized into oxylipins with pro- or anti-inflammatory properties. We detected several oxylipins in human sWAT, derived either from AA, DHA or EPA. The levels of most of these oxylipins did not differ between NGT and T2DM women, but there were tendencies for higher concentrations of some of the AA-derived leukotrienes (LTD_4_, 6-*trans* LTB_4_, and 6-*trans-*12-*epi* LTB_4_). For the leukotriene biosynthesis pathway (see Figure 4), AA is metabolized by 5-lipoxygenase (5-LOX) in the presence of an integral nuclear membrane protein, 5-LOX activating protein (also called FLAP). In this reaction, AA is metabolized into the sequential intermediates 5-hydroperoxyeicosatetraenoic acid (5-HpETE) and LTA_4_. LTA_4_ is conjugated with reduced glutathione by LTC_4_ synthase (LTC4S) and released from the cell for extracellular conversion to LTD_4_ and LTE_4_. Alternatively, cytosolic LTA_4_ hydrolase (LTA4H) converts LTA_4_ to LTB_4_. LTA_4_ is also hydrolyzed non-enzymatically into 6-*trans* LTB_4_ and 6-*trans*-12-*epi* LTB_4_. To further study the leukotriene biosynthesis pathway we analyzed gene expression of genes involved in this pathway. We found a significant higher expression of *ALOX5* (5-LOX), *ALOX5AP* (FLAP), and *DPEP2* (dipeptidase 2), indicating an overall up-regulation of the 5-LOX pathway in sWAT of T2DM women. The fact that both LTD_4_ and the non-enzymatically hydrolyzed LTB_4_’s tended to be higher (and not only one particular leukotriene) is in line with this notion. Subcutaneous adipose tissue expression of *ALOX5AP* has previously been shown to be positively associated to body weight and insulin resistance as determined by HOMA-IR index [27]. The 5-LOX pathway has also been shown to be increased in obese adipose tissue [28]. Mouse and human adipocytes produce leukotrienes *in vitro* and this production is increased in hypertrophic adipocytes in obesity in mice [29]. Taken together, previous studies and our study suggest that the 5-LOX pathway may provide a link between adipose tissue, inflammation, and insulin resistance.

Most of the studies on lipid signaling and oxylipins in adipose tissue have been performed in rats or mice. We have performed a study in humans and have made a thorough analysis of several types of oxylipins and concentrations thereof in adipose tissue. We did not detect resolvins in adipose tissue apart from 10*S*,17*S*-diHDHA (PDX) and in fewer than five women we could detect some Resolvin E2 and Maresin 1. This is in contrast with several mouse studies that detected more resolvins in adipose tissue and suggested a role for these mediators in counteracting adipose tissue inflammation [31,32,33]. Apart from the fact that there may be species specific differences between the studies it is also possible that discrepancies can be explained by the stage of adipose tissue inflammation studied. Most of the mouse studies examine adipose tissue inflammation during a high fat diet feeding, thus during progressive adipose tissue expansion and inflammation. Our obese women had been obese for a relatively long period of time and their adipose tissue inflammation was likely fairly established. Further research is required to determine whether species-specificity or the stage and duration of obesity are responsible for the observed differences. Additionally, the analysis of oxylipins in adipose tissue is a challenging task due to the large amounts of triglycerides and other hydrophobic matrix constituents. In the presented study we adapted a published protocol for the analysis of adipose tissue, however it has to be mentioned that the recoveries particularly for the internal standards 15-HETE-d8 and DHA-d5 were rather low (<40%), suggesting that an improvement of the described method for further studies might be of importance.

## 5. Conclusions

In conclusion, AA and DHA content were higher in sWAT of T2DM women and associated with the inflammatory state in the tissue. The increased AA content was accompanied by an up-regulation of the genes in the leukotriene pathway and this seems to have led to a modest increase in the conversion of AA into pro-inflammatory leukotrienes in the subcutaneous adipose tissue of individuals with T2DM. 

## Supplementary Files

Supplementary File 1
