# Supplementary material for: Increased PUFA Content and 5-Lipoxygenase Pathway Expression Are Associated with Subcutaneous Adipose Tissue Inflammation in Obese Women with Type 2 Diabetes
Source: Nutrients. 2015 Sep 11;7(9):7676–90. doi: 10.3390/nu7095362 (PMC4586557; doi:10.3390/nu7095362)
Supplement: Supplementary File 1 [file nutrients-07-05362-s001.docx]

**Supplementary Information**

**Table S1.** Multiple Reaction Monitoring setup for ion transitions of the target compounds. Symbols in black refer to internal standards. RT, retention time, Q1, quadrupole 1 ion selection, Q3, quadrupole 3 ion selection, EP, entrance potential, CE, collision energy, CCEP, collision cell exit potential. HODEs, HOTrEs, HETEs, HEPEs, diHETEs and diHDPAs are given without chiral descriptors.

| **Symbol** | **Lipid Maps ID** | **RT (min)** | **Q1  (*m*/*z*)** | **Q3  (*m*/*z*)** | **DP (Volts)** | **EP (Volts)** | **CE (Volts)** | **CCEP (Volts)** |
| --- | --- | --- | --- | --- | --- | --- | --- | --- |
| RvE1 | LMFA03070019 | 4.0 | 349.1 | 195.0 | −95 | −10 | −22 | −13 |
| 20-hydroxy LTB_4_ | LMFA03020018 | 4.4 | 351.1 | 195.0 | −60 | −10 | −24 | −17 |
| 8-*iso*-PGF_2_α | LMFA03110001 | 5.1 | 353.1 | 193.0 | −135 | −10 | −34 | −11 |
| 15-*keto*-PGE_2_ | LMFA03010030 | 5.1 | 349.0 | 234.9 | −65 | −10 | −20 | −13 |
| TxB_2_ | LMFA03030002 | 5.2 | 369.1 | 169.0 | −55 | −10 | −24 | −15 |
| 8-*iso*-PGE_2_ | LMFA03110003 | 5.3 | 351.1 | 271.0 | −5 | −10 | −24 | −19 |
| PGE_2_-d_4_ | LMFA03010008 | 5.6 | 355.1 | 193.0 | −50 | −10 | −26 | −17 |
| PGE_2_ | LMFA03010003 | 5.7 | 351.2 | 271.1 | −50 | −10 | −22 | −21 |
| PGD_2_ | LMFA03010004 | 5.8 | 351.1 | 233.0 | −30 | −10 | −16 | −13 |
| LXB_4_ | LMFA03040002 | 6.0 | 351.1 | 220.9 | −60 | −10 | −22 | −13 |
| PGF_2α_ | LMFA03010002 | 6.1 | 353.1 | 193.0 | −80 | −10 | −34 | −11 |
| RvD2 | LMFA04000007 | 6.2 | 375.1 | 277.1 | −60 | −10 | −18 | −15 |
| LXA_4_ | LMFA03040001 | 6.5 | 351.1 | 114.8 | −40 | −10 | −20 | −11 |
| 13,14-dihydro-15-keto-PGF2α | LMFA03010027 | 6.6 | 353.1 | 195.0 | −110 | −10 | −32 | −11 |
| AT-RvD1 | LMFA04000074 | 6.7 | 375.0 | 215.0 | −50 | −10 | −26 | −11 |
| RvD1 | LMFA04000006 | 6.7 | 375.1 | 215.0 | −50 | −10 | −26 | −11 |
| AT-LXA4 | LMFA03040003 | 6.8 | 351.1 | 114.9 | −20 | −10 | −22 | −11 |
| RvE2 | LMFA03070036 | 7.8 | 333.1 | 114.9 | −35 | −10 | −18 | −15 |
| 18S-RvE3 | LMFA03070048 | 8.8 | 333.1 | 245.2 | −25 | −10 | −16 | −17 |
| 6-trans-LTB4 | LMFA03020013 | 8.9 | 335.1 | 194.9 | −105 | −10 | −22 | −11 |
| 8S,15S-diHETE | LMFA03060050 | 8.9 | 335.1 | 207.9 | −55 | −10 | −22 | −17 |
| 5,15-diHETE | LMFA03060010 | 9.2 | `335.0 | 173.1 | −55 | −10 | −20 | −11 |
| 8(9)-EET | LMFA03080003 | 11.4 | 319.0 | 155.0 | −60 | −10 | −10 | −13 |
| 11(12)-EET | LMFA03080004 | 11.4 | 319.0 | 167.0 | −90 | −10 | −18 | −19 |
| 14(15)-EET | LMFA03080005 | 11.2 | 319.0 | 219 | −5 | −10 | −16 | −55 |
| LTD4 | LMFA03020006 | 9.0 | 495.1 | 177.0 | −70 | −10 | −28 | −19 |
| 6-trans-12-epi-LTB4 | LMFA03020014 | 9.1 | 335.1 | 194.9 | −80 | −10 | −22 | −25 |
| 10S,17S-diHDHA (PDX) | LMFA04000047 | 9.2 | 359.1 | 153.0 | −70 | −10 | −22 | −9 |
| 18R-RvE3 | LMFA03070049 | 9.2 | 333.1 | 245.0 | −55 | −10 | −18 | −23 |
| 7S-MaR1 | n.a. | 9.3 | 359.1 | 249.9 | −20 | −10 | −20 | −19 |
| MaR1 | LMFA04000048 | 9.4 | 359.2 | 250.2 | −65 | −10 | −20 | −13 |
| LTB4-d4 | LMFA03020030 | 9.4 | 339.1 | 196.9 | −70 | −10 | −22 | −19 |
| LTB4 | LMFA03020001 | 9.4 | 335.1 | 195.0 | −65 | −10 | −22 | −21 |
| 14,15-diHETE | LMFA03060077 | 9.5 | 335.1 | 207.0 | −65 | −10 | −24 | −21 |
| 7,17-diHDPA | n.a. | 9.5 | 361.1 | 198.9 | −45 | −10 | −26 | −23 |

**Table S1. *Cont.***

| **Symbol** | **Lipid Maps ID** | **RT (min)** | **Q1  (m/z)** | **Q3  (m/z)** | **DP (Volts)** | **EP (Volts)** | **CE (Volts)** | **CCEP (Volts)** |
| --- | --- | --- | --- | --- | --- | --- | --- | --- |
| LTE4 | LMFA03020002 | 9.6 | 438.1 | 333.1 | −55 | −10 | −26 | −15 |
| 19,20-diHDPA | LMFA04000043 | 10.2 | 361.1 | 273.0 | −55 | −10 | −22 | −15 |
| 9-HOTrE | LMFA02000024 | 10.2 | 293.0 | 170.9 | −75 | −10 | −20 | −15 |
| 13-HOTrE | LMFA02000051 | 10.3 | 293.0 | 195.0 | −45 | −10 | −24 | −19 |
| 18-HEPE | LMFA03070038 | 10.4 | 317.1 | 259.0 | −5 | −10 | −16 | −7 |
| 15-HEPE | LMFA03070009 | 10.5 | 317.1 | 219.0 | −65 | −10 | −18 | −19 |
| 13-HODE | LMFA02000228 | 10.8 | 295.0 | 194.9 | −110 | −10 | −24 | −21 |
| 9-HODE | LMFA02000188 | 10.8 | 295.0 | 171.0 | −130 | −10 | −22 | −7 |
| 15-HETE-d8 | LMFA03060080 | 10.9 | 327.2 | 226.0 | −85 | −10 | −18 | −11 |
| 15-HETE | LMFA03060001 | 11.0 | 319.1 | 219.1 | −55 | −10 | −18 | −9 |
| 11-HETE | LMFA03060003 | 11.1 | 319.1 | 167.0 | −70 | −10 | −22 | −15 |
| 7-HDHA | n.a. | 11.3 | 343.1 | 141.1 | −85 | −10 | −18 | −23 |
| 10-HDHA | n.a. | 11.2 | 343.1 | 153.0 | −25 | −10 | −20 | −15 |
| 17-HDHA | LMFA04000072 | 11.1 | 343.1 | 245.0 | −65 | −10 | −16 | −15 |
| 12-HETE | LMFA03060007 | 11.2 | 319.1 | 179.0 | −65 | −10 | −20 | −23 |
| 8-HETE | LMFA03060006 | 11.2 | 319.1 | 154.9 | −70 | −10 | −20 | −19 |
| 5-HETE | LMFA03060002 | 11.3 | 319.1 | 115.0 | −65 | −10 | −18 | −11 |
| ALA | LMFA01030152 | 12.4 | 277.0 | 233.0 | −90 | −10 | −22 | −29 |
| EPA | LMFA01030759 | 12.4 | 301.0 | 202.9 | −125 | −10 | −18 | −21 |
| DHA-d5 | LMFA01030762 | 12.4 | 332.0 | 288.1 | −75 | −10 | −16 | −13 |
| DHA | LMFA01030185 | 12.7 | 327.1 | 229.2 | −115 | −10 | −18 | −11 |
| AA | LMFA01030001 | 12.7 | 303.0 | 205.1 | −155 | −10 | −20 | −11 |
| LA | LMFA01030120 | 12.8 | 279.0 | 261.0 | −115 | −10 | −28 | −13 |
| DPA *n*-3 | LMFA04000044 | 13.0 | 329.1 | 231.1 | −50 | −10 | −20 | −17 |
| AdA | LMFA01030178 | 13.1 | 331.1 | 233.0 | −130 | −10 | −22 | −11 |


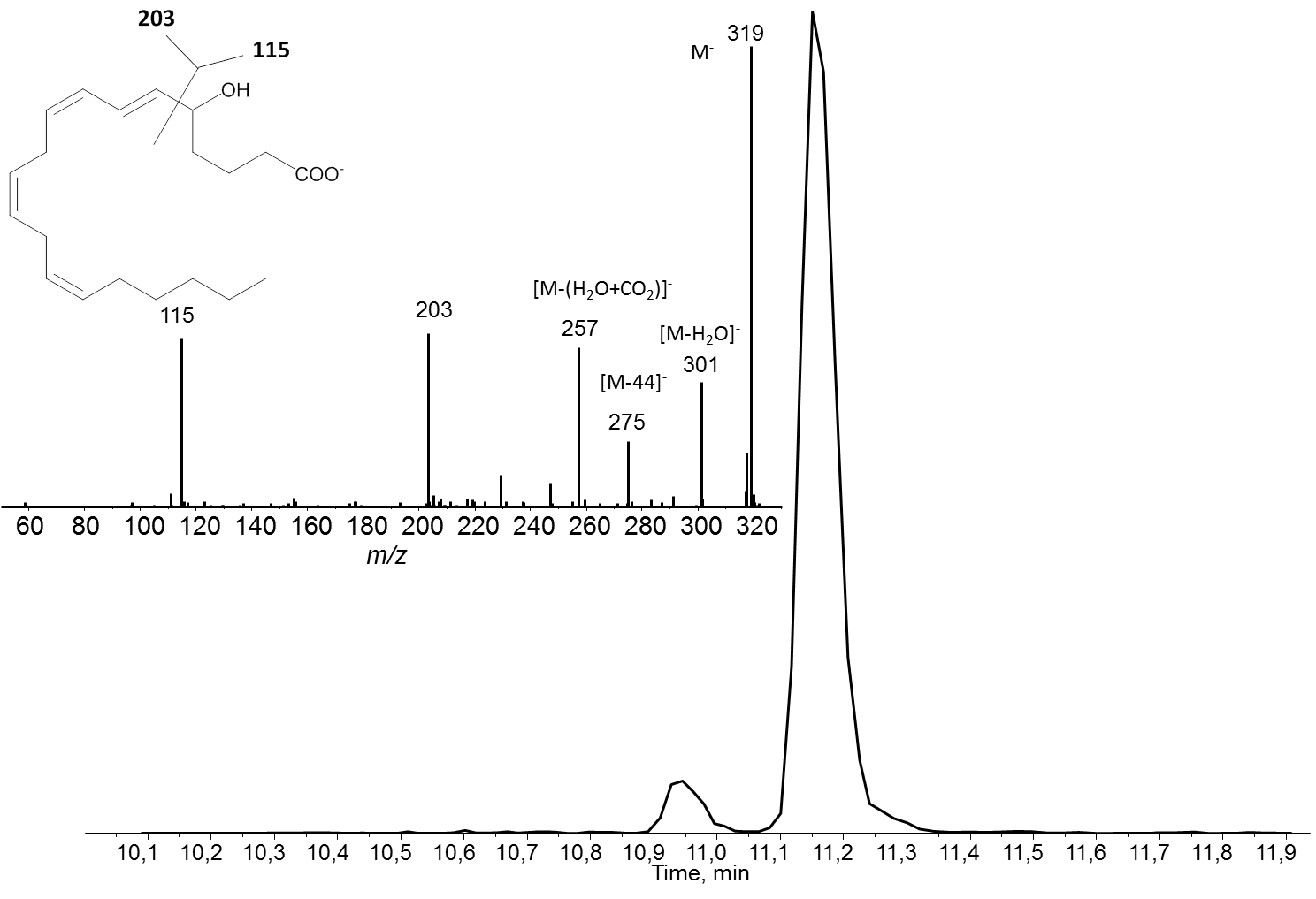


**Figure S1.** SRM transition *m*/*z* 319 → 115 showing 5-HETE at RT = 11.2 min and its two isomers. Upper left corner characteristic MS/MS spectrum and fragmentation of 5-HETE.


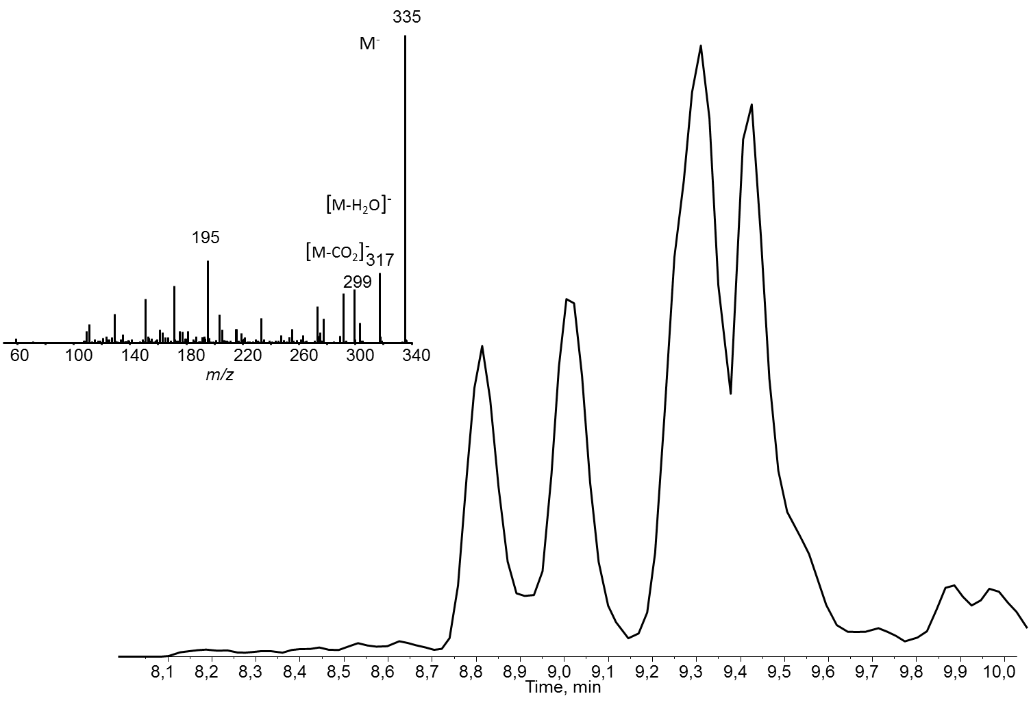


**Figure S2.** SRM transition *m*/*z* 335 → 195 showing LTB4 at RT = 9.3 min and its two isomers. Upper left corner characteristic MS/MS spectrum and fragmentation of LTB4.


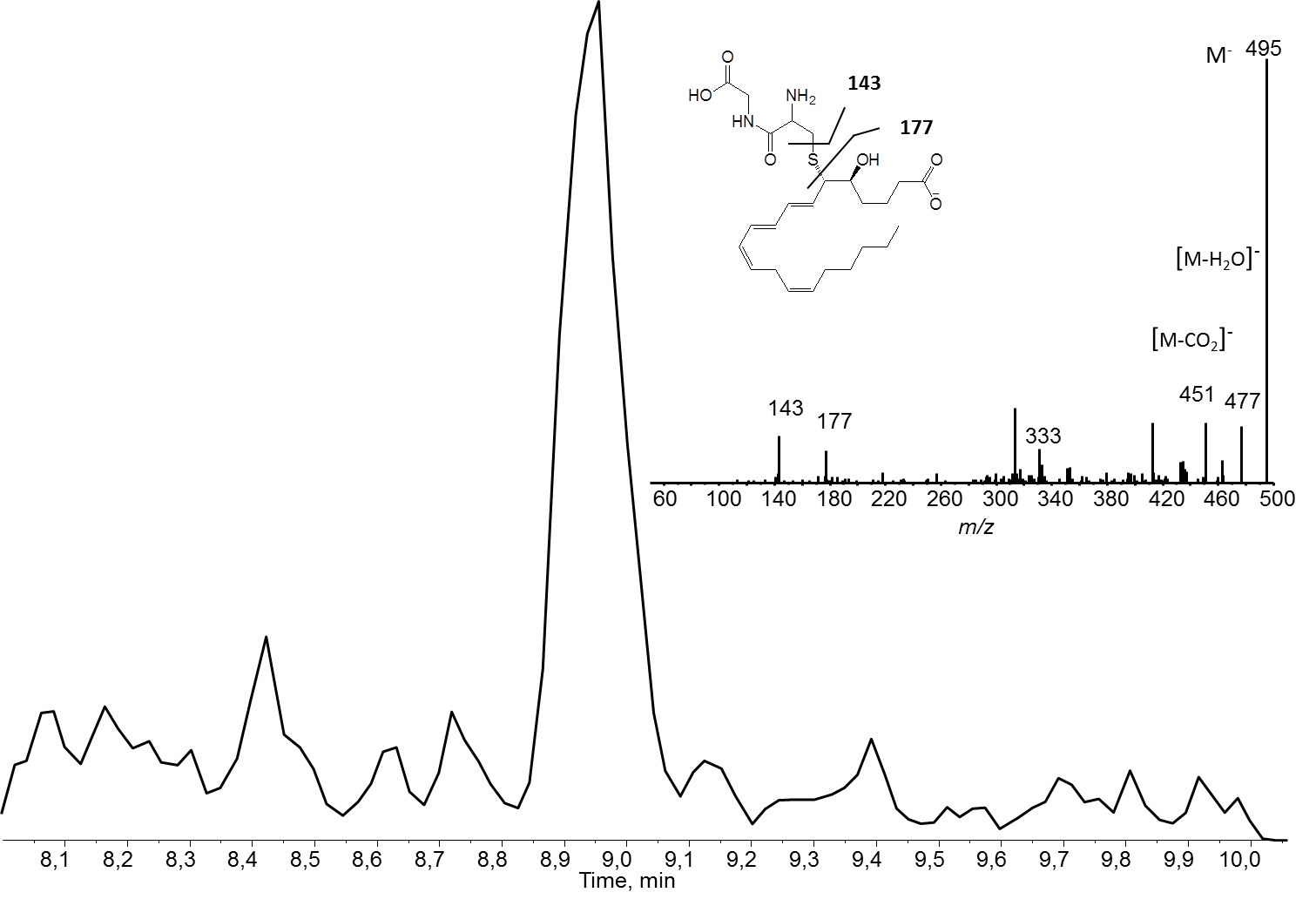


**Figure S3.** SRM transition *m*/*z* 495 → 177 showing LTD4 at RT = 8.9 min. Upper right corner characteristic MS/MS spectrum and fragmentation of LTD4.

© 2015 by the authors; licensee MDPI, Basel, Switzerland. This article is an open access article distributed under the terms and conditions of the Creative Commons Attribution license (http://creativecommons.org/licenses/by/4.0/).
